# NEAT1 Confers Radioresistance to Hepatocellular Carcinoma Cells by Inducing PINK1/Parkin-Mediated Mitophagy

**DOI:** 10.3390/ijms232214397

**Published:** 2022-11-19

**Authors:** Hiroyuki Tsuchiya, Ririko Shinonaga, Hiromi Sakaguchi, Yutaka Kitagawa, Kenji Yoshida, Goshi Shiota

**Affiliations:** 1 Division of Medical Genetics and Regenerative Medicine, Department of Genomic Medicine and Regenerative Therapeutics, Faculty of Medicine, Tottori University, 86 Nishi-cho, Yonago 683-8503, Japan; 2 Department of Radiation Oncology, Tottori University Hospital, 86 Nishi-cho, Yonago 683-8503, Japan

**Keywords:** NEAT1, mitophagy, radiation resistance, SOD2, GAPARAP, parkin, PINK1

## Abstract

A long noncoding RNA, nuclear paraspeckle assembly transcript 1 (NEAT1) variant 1 (NEAT1v1), confers radioresistance to hepatocellular carcinoma (HCC) cells by inducing autophagy via γ-aminobutyric acid A receptor-associated protein (GABARAP). Radiation induces oxidative stress to damage cellular components and organelles, but it remains unclear how NEAT1v1 protects HCC cells from radiation-induced oxidative stress via autophagy. To address this, we precisely investigated NEAT1v1-induced autophagy in irradiated HCC cell lines. X-ray irradiation significantly increased cellular and mitochondrial oxidative stress and mitochondrial DNA content in HCC cells while NEAT1v1 suppressed them. NEAT1v1 concomitantly induced the phosphatase and tensin homolog-induced kinase 1 (PINK1)/parkin-mediated mitophagy. Interestingly, parkin expression was constitutively upregulated in NEAT1v1-overexpressing HCC cells, leading to increased mitochondrial parkin levels. Superoxide dismutase 2 (SOD2) was also upregulated by NEAT1v1, and GABARAP or SOD2 knockdown in NEAT1v1-overexpressing cells increased mitochondrial oxidative stress and mitochondrial DNA content after irradiation. Moreover, it was suggested that SOD2 was involved in NEAT1v1-induced parkin expression, and that GABARAP promoted parkin degradation via mitophagy. This study highlights the unprecedented roles of NEAT1v1 in connecting radioresistance and mitophagy in HCC.

## 1. Introduction

The prognosis of patients with hepatocellular carcinoma (HCC), ~80% of primary liver cancers, is poor because therapeutic options for HCC are limited due to the increased levels of the carcinogenic potential of coexisting chronic liver diseases [1]. Several molecular-targeted drugs and immune checkpoint inhibitors have recently been introduced for treating HCC; however, those agents are expected to prolong patient survival by only several months [2]. Therefore, the continuous challenge to develop more efficient therapeutic options is required to improve the prognosis of patients with HCC.

Radiation therapy is minimally invasive, and targets selective treatment compared to surgery and chemotherapy [3]. Moreover, recent advancements in radiation therapy, such as stereotactic body radiation therapy and heavy ion radiotherapy, efficiently deliver an ablative radiation dose to tumors [3,4,5]. However, local recurrence remains an important issue of radiation therapy for HCC.

Nuclear paraspeckle assembly transcript 1 (NEAT1) is a long noncoding RNA (lncRNA) expressed in shorter (NEAT1v1) and longer (NEAT1v2) isoforms [6,7]. NEAT1v1 is required for the maintenance and induction of cancer stem cells (CSCs), endowed with a tumor-initiating property, in HCC cells [8,9]. Moreover, consistent with the therapeutic resistant features of CSCs [9], NEAT1v1 also confers radioresistance to HCC cells [10]. By performing an autophagic flux assay, we previously demonstrated that NEAT1v1 enhances autophagy in irradiated cells via γ-aminobutyric acid A receptor-associated protein (GABARAP): a critical factor for autophagosome-lysosome fusion during starvation-induced autophagy and phosphatase and tensin homolog-induced kinase 1 (PINK1)/parkin-mediated mitophagy [11,12].

Autophagy is a critical process for maintaining cellular homeostasis; it can be tumorigenic and tumor-suppressive depending on the biological context [13,14,15,16,17]. Radiation generates a massive amount of reactive oxygen species (ROS) in cancer cells, which irreversibly damages cellular organelles and biomolecules [18]. In this context, autophagy protects cancer cells from radiation by promoting the regeneration of damaged organelles. Autophagy also controls the mitochondrial quality by degrading mitochondria in nonselective (macroautophagy) and selective (mitophagy) manners. Mitophagy selectively targets damaged mitochondria to maintain mitochondrial homeostasis [19]. Mitophagy induction involves several pathways, including ubiquitin-dependent and -independent receptor pathways [19]. The former is also recognized as the PINK1/parkin pathway. Under normal conditions, PINK1 translocates to the inner membrane of healthy mitochondria and is proteolytically cleaved by mitochondrial proteases [20,21]. Cleaved PINK1 is retrotranslocated to the cytosol and subjected to proteasomal degradation [19]. However, upon mitochondrial depolarization, the translocation is inhibited, and PINK1 is stabilized and exposed to the outer mitochondrial membrane [20,21]. PINK1 on the outer membrane phosphorylates the Ser65 residue of ubiquitin and parkin, thereby activating the E3 ubiquitin ligase activity of parkin [22,23]. Activated parkin accumulates on the outer membrane and induces the K63 ubiquitination of mitochondrial proteins [22,23]. These ubiquitinated proteins are recognized by autophagy cargo receptors, such as optineurin and NDP52, which also bind to GABARAP and LC3 on the autophagosomal membrane, resulting in the engulfment of damaged mitochondria [23,24]. GABARAP and LC3 are members of the ATG8 family and share structural similarities and redundant functions. However, GABARAP is more responsible for the PINK1/parkin-mediated mitophagy than LC3 because the GABARAP knockout markedly impairs the PINK1/parkin-mediated mitophagy, whereas no effect is observed in LC3 knockout cells [12].

This study investigated the effects of NEAT1v1 on mitophagy in irradiated HCC cells and found that NEAT1v1 induces PINK1/parkin-mediated mitophagy via GABARAP and superoxide dismutase 2 (SOD2) to protect HCC cells from radiation.

## 2. Results

### 2.1. NEAT1v1 Suppresses Radiation-Induced Mitochondrial Damage

**Figure 1:**
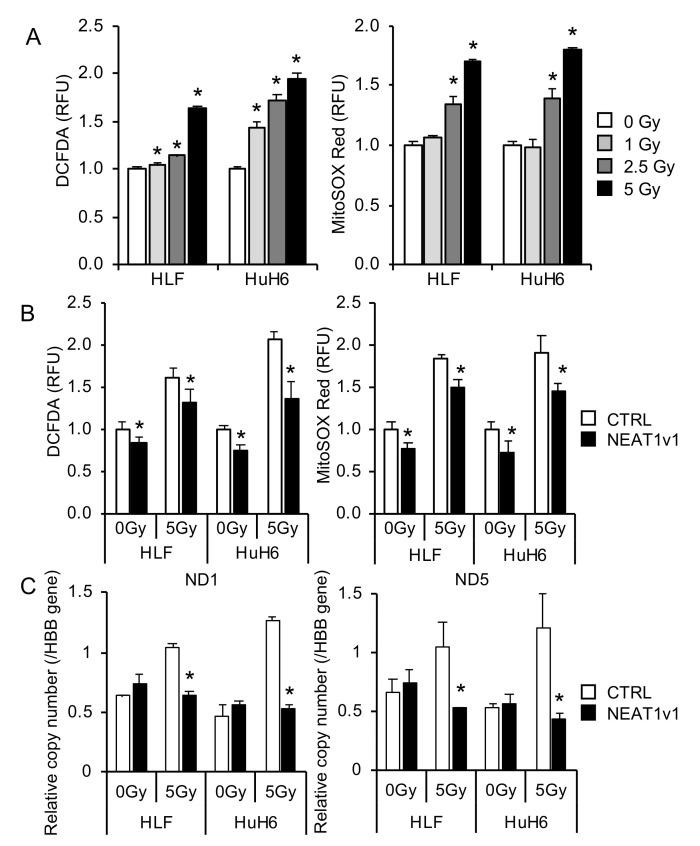
Protective effects of NEAT1v1 on radiation-induced mitochondrial damage. (**A**) Radiation dose-dependent increases in cellular (left) and mitochondrial (right) oxidative stress. * *p* < 0.05 (Dunnett’s test vs. 0 Gy; *n* = 4). (**B**) Suppression of radiation-induced cellular (left) and mitochondrial (right) oxidative stress. * *p* < 0.05 [Student’s *t*-test, control (CTRL) vs. NEAT1v1-overexpressing cells; *n* = 4]. (**C**) Relative copy number of mitochondrially encoded genes (*ND1* and *ND5*). * *p* < 0.05 (Student’s *t*-test, CTRL vs. NEAT1v1-overexpressing cells; *n* = 3).

Radiation induces cellular damage by exacerbating oxidative stress. Cellular and mitochondrial oxidative stress were examined with DCFDA and MitoSOX Red, respectively, in irradiated HCC cells. In Figure 1A, cellular and mitochondrial oxidative stress significantly increased by irradiation in a dose-dependent manner. In contrast, oxidative stress was significantly suppressed by NEAT1v1 (Figure 1B). Moreover, the relative copy numbers of mitochondrially encoded genes, *ND1* and *ND5*, to the nuclear-encoded *HBB* gene markedly increased by radiation in control HCC cell lines (Figure 1C). In contrast, this increase was abolished by NEAT1v1 (Figure 1C). The determination of the copy number of *ND1* and *ND5* genes relative to a nuclear gene are an established method to estimate the mitochondrial DNA copy number in a cell [25,26]. These results indicate that radiation induced the accumulation of mitochondrial DNA, while NEAT1v1 suppressed it. In summary, NEAT1v1 protected HCC cells by suppressing radiation-induced mitochondrial damage and the accumulation of damaged mitochondria.

### 2.2. NEAT1v1 Promotes Mitophagy in Irradiated HCC Cells

**Figure 2:**
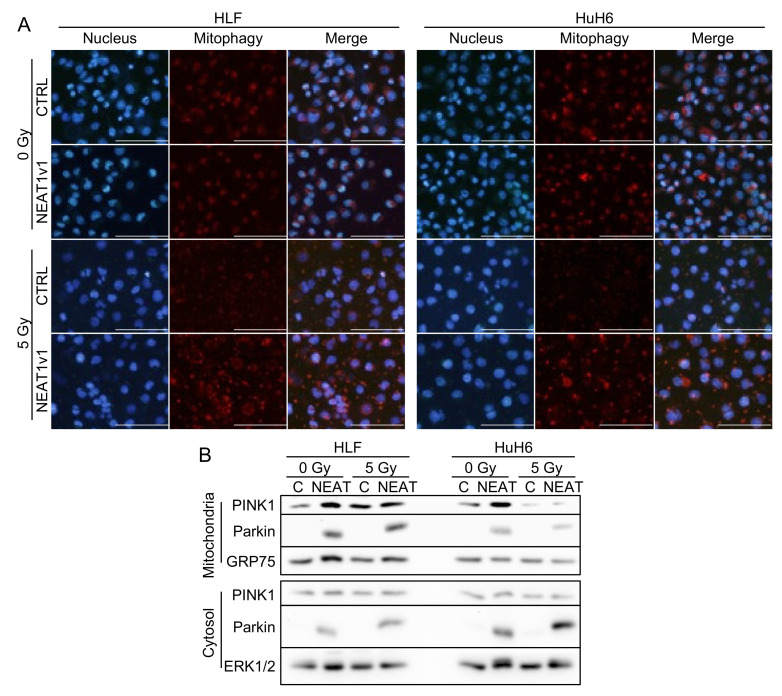
Induction of PINK1/parkin-mediated mitophagy by NEAT1v1 in irradiated cells. (**A**) Representative images of mitophagy staining. Mitophagy was stained with Mitophagy Dye (red). Nuclei were counterstained with Hoechst (blue). Scale bar, 100 µm. (**B**) Representative Western blot images for mitochondrial and cytosolic PINK1, parkin, GRP75 (mitochondrial marker), and ERK1/2 (cytosolic marker). C, CTRL; NEAT, NEAT1v1-overexpressing cells.

Because NEAT1v1 protects HCC cells from radiation by promoting autophagy [10], it was postulated that mitophagy might be involved in this phenomenon. Indeed, LC3 localization in mitochondria was observed in irradiated HCC cells (Figure S1). Mitophagy staining showed that similar levels of mitophagy occurred between the control and NEAT1-overexpressing cells in a nonirradiated condition (Figure 2A). However, after irradiation, mitophagy in the control cells tended to be impaired, compared with the NEAT1v1-overexpressing cells (Figure 2A and Figure S2).

In nonirradiated cells, NEAT1v1 induced parkin expression in mitochondria and cytosol, whereas mitochondrial localization of PINK1 increased (Figure 2B). Mitochondrial and cytosolic parkin remained high in NEAT1v1-overexpressing cells after irradiation, whereas mitochondrial PINK1 levels were similar between the control and NEAT1v1-overexpressing cells (Figure 2B). These results suggested that NEAT1v1 enhanced mitochondrial localization of PINK1 in a nonirradiated condition while constitutively upregulating parkin to promote mitophagy.

### 2.3. SOD2 Is Involved in NEAT1v1-Induced Radioresistance

**Figure 3:**
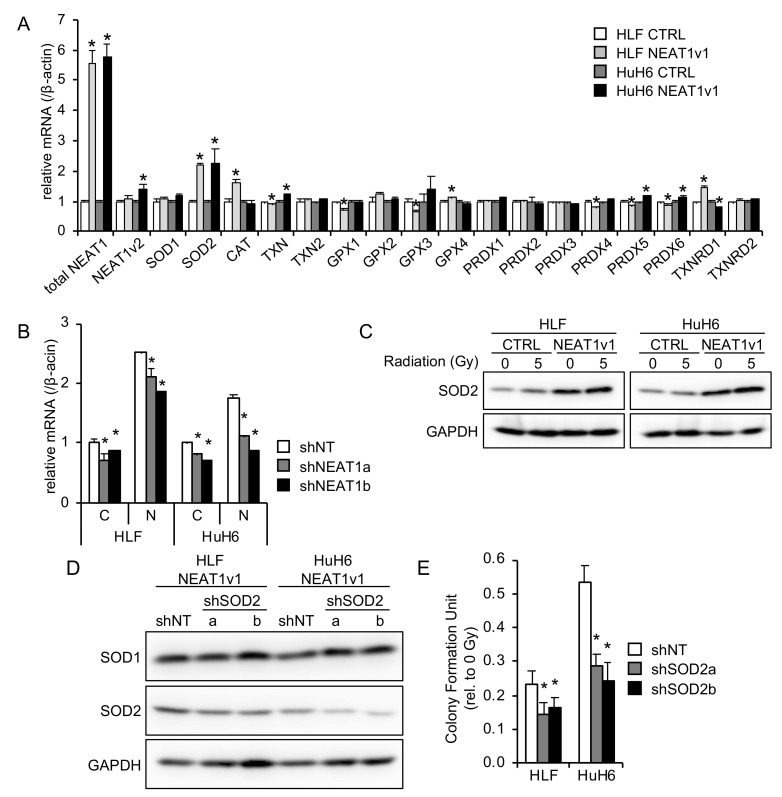
Involvement of SOD2 in NEAT1v1-induced radioresistance. (**A**) MRNA expression levels of genes encoding antioxidative enzymes in NEAT1v1-overexpressing cells. * *p* < 0.05 [Student’s *t*-test, control (CTRL) vs. NEAT1v1-overexpressing cells; *n* = 3]. (**B**) Expression levels of SOD2 mRNA in control (C) or NEAT1v1-overexpressing cells (N) transduced with adenoviruses expressing nontarget shRNA (shNT) or NEAT1-specific shRNAs (shNEAT1a and shNEAT1b). * *p* < 0.05 (Dunnett’s test vs. shNT; *n* = 3). (**C**) Representative Western blot images for SOD2 and GAPDH (internal control) using whole-cell lysates after 0 or 5 Gy irradiation. (**D**) Representative Western blot images for SOD1, SOD2, and GAPDH (internal control) using whole-cell lysates of cells transduced with adenoviruses expressing shNT or SOD2-specific shRNAs (shSOD2a and shSOD2b). (**E**) Colony formation abilities of NEAT1v1-overexpressing cells knocked down for SOD2 after 2.5 Gy irradiation. * *p* < 0.05 (Dunnett’s test vs. shNT; *n* = 6).

The suppression of oxidative stress by NEAT1 suggests an enhanced removal of ROS by antioxidative enzymes; thus, the expression of antioxidative enzymes was examined in NEAT1v1-overexpressing HCC cells. Both cell lines showed a significant increase in SOD2 expression by NEAT1v1, whereas other antioxidative enzymes showed no difference or significant changes in either cell line (Figure 3A). Because superoxide anion (O_2_^−^) is constitutively produced by the electron transfer reaction, mitochondria are one of the major sources of ROS, and SOD2 is a mitochondrial antioxidative enzyme that detoxifies O_2_^−^. Therefore, this result suggested that SOD2 was responsible for suppressing radiation-induced mitochondrial oxidative stress by NEAT1v1.

NEAT1 knockdown significantly downregulated SOD2 expression in the control and NEAT1v1-overexpressing HCC cells (Figure 3B and Figure S3), suggesting that NEAT1v1 directly regulated SOD2 mRNA expression. SOD2 protein was also upregulated by NEAT1v1 overexpression regardless of irradiation (Figure 3C). Because NEAT1v1 enhances radioresistance in HCC cells via GABARAP [10], the effects of SOD2 knockdown on NEAT1v1-induced radioresistance were further examined. ShRNAs targeting SOD2 downregulated SOD2 protein expression in NEAT1v1-overexpressing HCC cells but did not affect SOD1 expression (Figure 3D). The colony formation assay revealed that SOD2 knockdown significantly decreased the radioresistance of NEAT1v1-overexpressing HCC cells (Figure 3E). These results suggested that NEAT1v1 conferred radioresistance to HCC cells via SOD2 in addition to GABARAP.

### 2.4. GABARAP and SOD2 Suppress Radiation-Induced Mitochondrial Oxidative Stress

**Figure 4:**
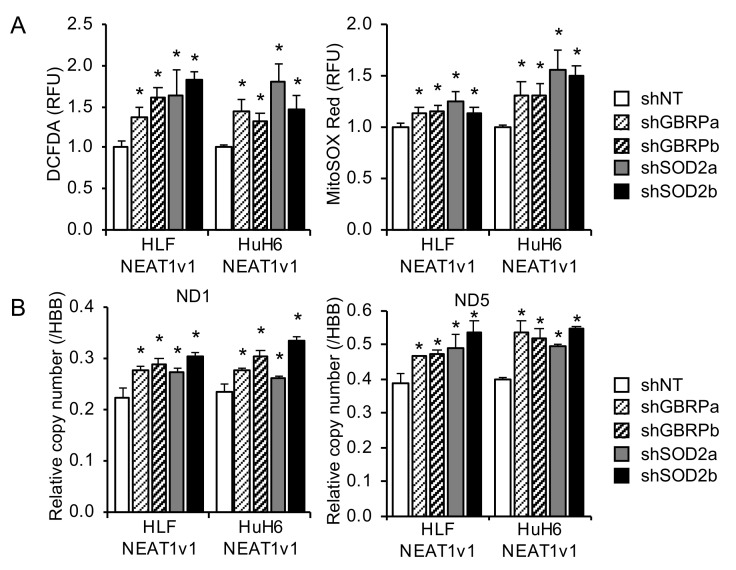
Involvement of GABARAP and SOD2 in the suppressive effects of NEAT1v1 on radiation-induced mitochondrial damage. (**A**,**B**) Cellular (left) and mitochondrial (right) oxidative stress (**A**) and relative copy number of mitochondrially encoded genes (ND1 and ND5); (**B**) in NEAT1v1-overexpressing HLF and HuH6 cells knocked down for GABARAP (shGBRPa and shGBRPb) or SOD2 (shSOD2a and shSOD2b) after 5 Gy irradiation. * *p* < 0.05 [Dunnett’s test vs. shNT; *n* = 6 (**A**) and 3 (**B**)].

The effects of the GABARAP and SOD2 knockdown on oxidative stress in HCC cells overexpressing NEAT1v1 after irradiation were investigated. In Figure 4A, cellular and mitochondrial oxidative stress after irradiation significantly increased by the GABARAP and SOD2 knockdown. These results suggested that GABARAP and SOD2 were involved in the NEAT1v1-mediated protection of HCC cells from radiation-induced oxidative stress. Concomitantly, the knockdown significantly increased the mitochondrial DNA copy number (Figure 4B).

### 2.5. GABARAP and SOD2 Are Involved in NEAT1v1-Induced Mitophagy

**Figure 5:**
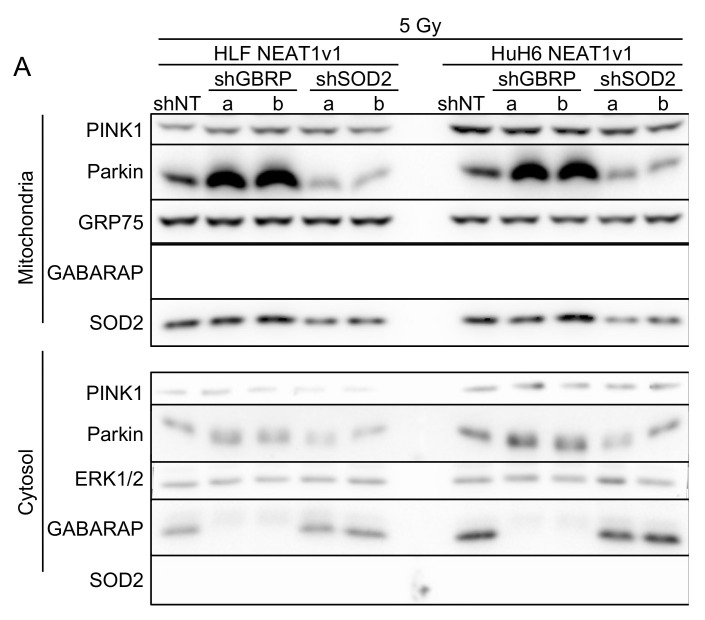

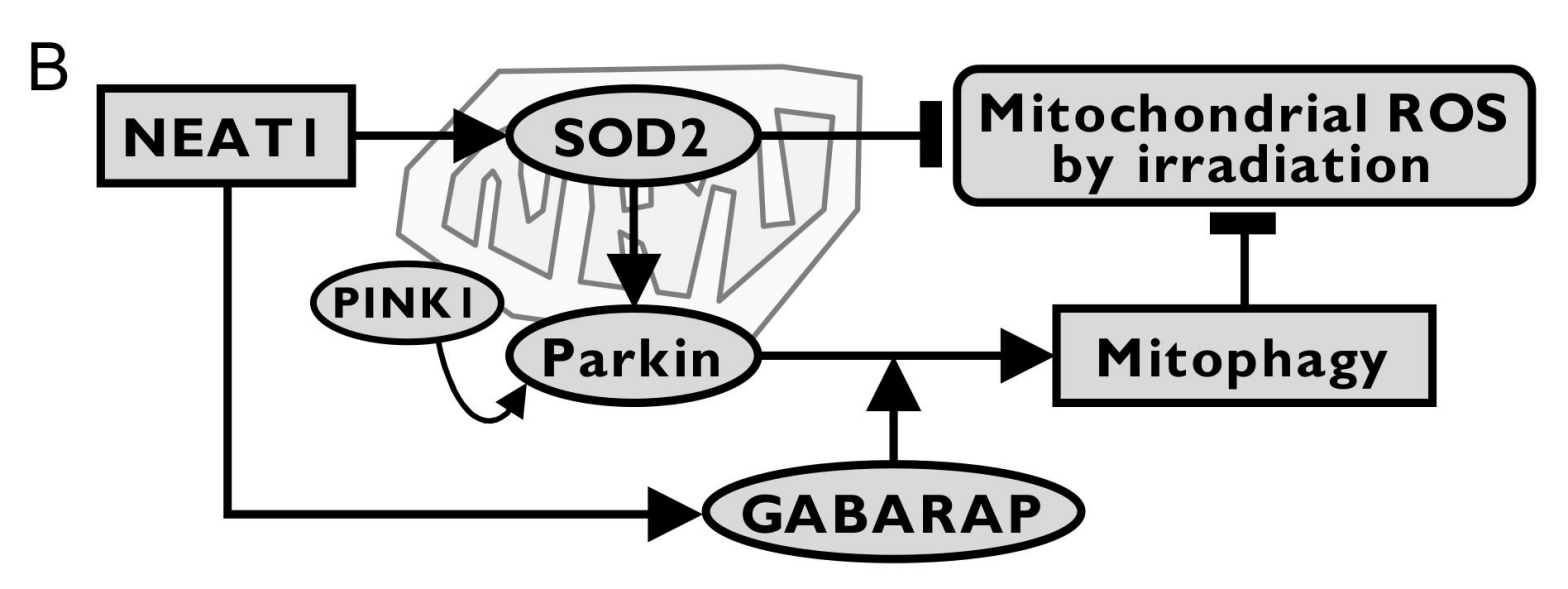
Involvement of GABARAP and SOD2 in NEAT1v1-induced mitophagy. (**A**) Representative Western blot images for mitochondrial and cytosolic PINK1, parkin, SOD2, GABARAP, GRP75 (mitochondrial marker), and ERK1/2 (cytosolic marker). C, CTRL, control cells; NEAT, NEAT1v1-overexpressing cells. (**B**) Schematic representation of NEAT1v1-induced radioresistance via the PINK1/parkin-mediated mitophagy. NEAT1v1 upregulates GABARAP and SOD2 in HCC cells. GABARAP is a critical factor for mitophagy, whereas SOD2 reduces oxidative stress by its antioxidative activity and induces parkin expression.

GABARAP is a critical factor for mitophagy [11,12], and SOD2 is a mitochondria-specific antioxidative enzyme. Moreover, their knockdown induced the accumulation of damaged mitochondria (Figure 4), suggesting that these two factors played a role in NEAT1v1-induced mitophagy. Whereas the GABARAP and SOD2 knockdown in NEAT1v1-overexpressing cells did not affect PINK1 expression, parkin expression markedly increased by the GABARAP knockdown (Figure 5A), consistent with a previous report, in which the GABARAP knockdown perturbed autophagy [10]. In contrast, the SOD2 knockdown resulted in parkin downregulation in the cytosol and mitochondria (Figure 5A), suggesting that SOD2 was involved in the constitutive upregulation of parkin by NEAT1v1.

## 3. Discussion

This study demonstrated that radiation increases mitochondrial oxidative stress, whereas NEAT1v1 suppresses it by enhancing PINK1/parkin-mediated mitophagy through GABARAP and SOD2 (Figure 5B). Considering that NEAT1v1 confers radioresistance to HCC cells [10], NEAT1v1 promotes the regeneration of healthy mitochondria by removing damaged mitochondria through mitophagy. Moreover, it was shown that NEAT1v1 significantly suppressed oxidative stress (Figure 1B), possibly due to the increased expression of SOD2, and increased the expression and mitochondrial localization of parkin (Figure 2B) even in nonirradiated cells. These findings suggest that NEAT1v1 regulates the basal levels of mitophagy in HCC cells under a physiological condition. This is in agreement with our previous report [10], in which NEAT1v1 was shown to promote autophagy in nonirradiated HCC cells. However, mitophagy staining using Mitophagy Dye showed no difference between the control and NEAT1v1-overexpressing cells without irradiation (Figure 2A). Although other mitophagy assessments, e.g., those using mito-OC [27] or mito-Keima [28], would more accurately quantify their difference, the contribution of NEAT1v1 to basal mitophagy may not be so significant, at least, to the extent that Mitophagy Dye can detect. Moreover, GABARAP or SOD2 knockdown resulted in a marginal increase in mitochondrial DNA, compared with the suppressive effect of NEAT1v1 on the radiation-induced accumulation of mitochondrial DNA (Figure 1C and Figure 4B). This might be due to the insufficient knockdown efficiency, in particular, of SOD2; however, it is known that the quality of mitochondria is controlled by several pathways, including ubiquitin-independent mitophagy and fission/fusion [29]. Therefore, it is possible that NEAT1v1 might also control the quality of mitochondria via such mechanisms other than the PINK1/parkin-mediated mitophagy. The comprehensive understanding of molecular mechanisms underlying NEATv1-mediated mitochondrial quality control should be addressed in future studies.

It was previously demonstrated that radiation suppressed autophagy [10]. This study also revealed increased mitochondrial DNA content and oxidative stress, suggesting the accumulation of damaged mitochondria in control cells after irradiation at a dose of 5 Gy. Consistently, a low dose (0.5 Gy) of carbon ions modestly damaged mitochondria and induced mitophagy in cervical and breast cancer cells [30]. However, when treated with a high dose (3 Gy), apoptosis was preferentially induced rather than mitophagy [30]. Because the content of mitochondrial DNA after irradiation was not increased in NEAT1v1-overexpressing cells, NEAT1v1 can promote the removal of damaged mitochondria through mitophagy even at the lethal irradiation dose. In the present study, we assessed mitophagy at 48 h after irradiation based on our preliminary experiments, in which an apparent difference in mitophagy staining was observed at 48 h, but not at 24 h after irradiation. Radiation-induced cell death via mitotic catastrophe requires a relatively long period because it must be preceded by several attempted divisions to accumulate sufficient genetic damage for mitotic death [31]. Therefore, it is worth investigating whether mitophagy induced by NEAT1v1 could contribute to cell survival at longer periods of culture time after irradiation.

NEAT1v1-induced autophagy has a cytoprotective effect on cancer cells [10]. Consistently, NEAT1 also induces autophagy by targeting miR-34a and miR-204 as a competing endogenous RNA (ceRNA) in colorectal cancer and HCC, respectively, resulting in the upregulation of autophagy-related proteins ATG9A, ATG4A, and ATG3 [32,33]. Eventually, NEAT1-induced autophagy leads to chemoresistance to 5-fluorouracil and sorafenib [32,33]. Moreover, NEAT1 also increases PINK1 and parkin expression, thereby enhancing lung mitophagy in chronic obstructive pulmonary disease [34]. However, the mitophagy induction by NEAT1 in cancer cells has not been studied. In contrast, NEAT1 suppresses mitophagy in neuron and renal epithelial cells and exaggerates the pathogenesis of Alzheimer’s disease and diabetic nephropathy [35,36]. This inconsistency might arise from the difference between nontransformed and transformed cells. However, in mitophagy suppression, NEAT1 plays a role as a ceRNA against miR-150-5p [36]. Therefore, NEAT1v1 might also regulate GABARAP and SOD2 expression as a ceRNA.

It is worth noting that mRNA expression levels of other GABARAP subfamily members, GABARAPL1 and GABARAPL2, did not significantly change in NEAT1v1-overexpressing cells. Therefore, among the GABARAP subfamily, GABARAP plays a central role in NEAT1v1-induced mitophagy. However, GABARAP has been suggested as a tumor promoter and suppressor. Carcinogen-induced tumor incidence was significantly reduced in GABARAP-deficient mice [37]. Moreover, high GABARAP expression in tumor tissues was significantly associated with poor prognosis of patients with colorectal carcinoma and breast cancer [38,39]. Likewise, GABARAP was upregulated in tumor necrosis factor-α-resistant breast cancer cells, concomitant with several other autophagy-related genes [40]. In contrast, GABARAP suppressed breast cancer progression through the AKT/mTOR signaling pathway [41]; however, how GABARAP suppressed the signaling pathway remains unclear. This study is the first report demonstrating the involvement of GABARAP-induced mitophagy in the radioresistance of cancer cells. Further studies must be undertaken to clarify the clinical significance of GABARAP-induced mitophagy in tumors and radiotherapy.

The mitochondrial electron transfer chain generates O_2_^−^, which is catabolized to less toxic hydrogen peroxide by the well-known mitochondrial antioxidative enzyme, SOD2. Thus, SOD2 upregulation by NEAT1v1 protects HCC cells from mitochondrial oxidative stress. However, because mitophagy is induced by depolarization of the mitochondrial membrane potential [42,43,44,45], it is suggested that SOD2 inhibits mitophagy [46,47,48], although limited information is available on their direct relationship. In contrast, SOD2 activity was significantly reduced in the myocardium of aged mice, whereas mitophagy was concomitantly impaired [49]. Likewise, SOD2 activity and PINK1 expression concomitantly increased in the liver of Per-Arnt-Sim kinase-deficient mice under fasting conditions, in which increased mitophagy was suggested by a characteristic mitochondria morphology [50]. A similar result was also observed in human umbilical vein endothelial cells treated with a plant-derived substance, scutellarin, by which SOD2, parkin, and PINK1 expression were induced, concomitant with increased mitophagy [51]. Moreover, nuclear factor erythroid 2-related factor 2 (NRF2) regulates the expression of antioxidative enzymes, including SOD2, and mitophagy-related proteins, including p62 and PINK1, whereas parkin overexpression activates NRF2, leading to the upregulation of antioxidative enzymes, including SOD2 [52,53,54]. These observations suggest that SOD2 can be involved in mitophagy induction depending on a cellular context. It remains yet to be clarified how NEAT1v1 induces parkin expression via SOD2; however, direct evidence was provided for the involvement of SOD2 in mitophagy induction, as SOD2 knockdown significantly impaired NEAT1v1-induced mitophagy and downregulated parkin expression. These findings indicate that SOD2 is involved in the mitophagy induction in HCC cells by inducing parkin expression. In the future, it is necessary to clarify how SOD2 induces parkin expression and mitophagy in irradiated HCC cells.

The present study based on in vitro experiments demonstrated the protumor roles of SOD2 in HCC. Moreover, we recently found that SOD2 is involved in NEAT1v1-induced chemoresistance in HCC cells. Nonetheless, the pathological significance of SOD2 in HCC is under debate, as its expression showed different expression patterns between cohorts [55]. Consistently, it has been suggested that SOD2 has dual roles in cancer including HCC [56]. Therefore, it is of interest to investigate in vivo whether an SOD2-targeting therapy is a promising strategy to potentiate the therapeutic efficacy of chemo/radiotherapy against HCC.

Several mechanisms have been suggested to underlie the radiosensitivity/radioresistance of HCC, including ferroptosis [57], CD133+CSCs [58], autophagy induction [10], suppression of DNA repair by melatonin-induced lncRNA RAD51-AS1 [59], miR320b/RAD21 axis [60], etc. However, the involvement of mitophagy in radioresistance in HCC has not been reported; thus, this report highlights mitophagy as an important therapeutic target for HCC radiotherapy. Several studies also have suggested that mitophagy is a critical cellular process for determining the radiosensitivity of cancer cells other than HCC. Increased mitochondrial oxidative stress by radiation induced BNIP3- and BNIP3L-mediated mitophagy to protect colorectal cancer cells from radiation-induced cytotoxicity [61]. Moreover, a newly synthesized anticancer compound, temozolomide-perillyl alcohol conjugate, was shown to inhibit mitophagy, thereby sensitizing non-small lung cancer cell lines to radiation [62]. Therefore, the suppression of mitophagy is a promising strategy to improve the clinical efficacy of radiotherapy.

## 4. Materials and Methods

### 4.1. Cell Culture

HCC cell lines (HLF and HuH6) and those overexpressing human NEAT1v1 and their control cells have been reported previously [10]. At 24 h after seeding, cells were irradiated (0, 1, 2.5, or 5 Gy) using an X-ray generator (MX-160Labo; mediXtec Japan, Chiba, Japan).

### 4.2. Adenovirus Vectors

Adenovirus vectors expressing nontargeting (NT) short hairpin RNA (shRNA; shNT), NEAT1-targeting shRNAs (shNEAT1a/b), and GABARAP-targeting shRNAs (shGBRPa/b) have been reported previously [10].

Adenovirus vectors expressing SOD2-targeting shRNAs (shSOD2a/b) were constructed as reported previously [8]. Briefly, oligo DNAs (Table S1) were ligated into BsaI-digested pENTR/U6-AmCyan1 with Ligation High version 2 (Toyobo, Osaka, Japan). Then, shRNA and AmCyan1-expressing cassettes were transferred by the LR reaction to pAd/BLOCK-iT-DEST (Thermo Fisher Scientific, Waltham, MA, USA). Adenovirus vectors were constructed by transfection of PacI-digested adenovirus plasmid DNA with LipofectAMINE2000 into 293A cells (Thermo Fisher Scientific, Waltham, MA, USA) according to the manufacturer’s protocol. Adenovirus titer was determined by the infectious genome titration protocol [63]. When knocking down genes in irradiated cells, these adenoviruses were transduced immediately after irradiation.

### 4.3. Reverse Transcription-Quantitative Polymerase Chain Reaction (RT-qPCR) and Western Blot Analysis

An RT-qPCR and Western blot analysis were performed as reported previously [8,10]. mRNA and protein samples were prepared 48 h after seeding, adenovirus transduction, or irradiation. The primers used for the RT-qPCR are summarized in Table S1. β-Actin was used as an internal control for calculating the relative mRNA expression levels. Antibodies against 75-kDa glucose-regulated protein (GRP75; sc-133137), extracellular signal-regulated kinases (ERK) 1/2 (sc-514302), GABARAP (sc-377300), glyceraldehyde 3-phosphate dehydrogenase (GAPDH; sc-365062), parkin (sc-32282), PINK1 (sc-518052), SOD1 (sc-101523), and SOD2 (sc-133134) were purchased from Santa Cruz Biotechnology (Santa Cruz, CA, USA).

### 4.4. Preparation of Cytosolic and Mitochondrial Fractions

Cytosol and mitochondria were fractionated by the Cell Fractionation Kit-Standard (Abcam, Cambridge, MA, USA) according to the manufacturer’s protocol with a slight modification. In brief, 2 × 10^6^ cells at 48 h after irradiation were suspended in 300 µL of buffer A, and were combined with an equal volume of buffer B. The cells were rotated at room temperature for 7 min. Following centrifugation at 5000× *g* for 2 min, supernatants were transferred in new tubes, and were centrifuged again at 10,000× *g* for 2 min. The supernatants (cytosolic fractions) were recovered and stored −80 °C until use, while the pellets of two centrifugation steps were combined, and resuspended in 300 µL of buffer A. An equal volume of buffer C was added and rotated at room temperature for 10 min. Following centrifugation at 5000× *g* for 2 min, supernatants were transferred in new tubes, and were centrifuged again at 10,000× *g* for 2 min. The supernatants (mitochondrial fractions) were recovered and stored −80 °C until use.

### 4.5. Detemination of Mitochondrial DNA Content

Cells were recovered 48 h after irradiation and lysed in proteinase K buffer [500 mM KCl, 100 mM Tris-HCl (pH 8.0), 1% Tween 20, and 1 mg/mL proteinase K] for 3 h at 55 °C. After phenol/CHCl_3_ extraction, cellular DNA was precipitated with isopropanol and dissolved in H_2_O. A qPCR was performed to obtain the Ct values of mitochondrially encoded *NADH dehydrogenase subunit 1* (*ND1*) and *5* (*ND5*) genes and *nuclear-encoded hemoglobin subunit β* (*HBB*) gene with the primers in Table S1. Relative copy numbers were calculated from a standard curve created using serially diluted samples, and those of *ND1* and *ND5* genes were normalized by that of the *HBB* gene.

### 4.6. Mesurement of Oxidative Stress

Cells were recovered and counted 48 h after irradiation and incubated with 20 µM 2’,7’-dichlorofluorescein diacetate (DCFDA; DCFDA/H2DCFDA-Cellular ROS Assay Kit; Abcam, Cambridge, MA, USA) or 5 µM MitoSOX Red (Thermo Fisher Scientific, Waltham, MA, USA) for 30 min at 37 °C. Cells were washed once with phosphate-buffered saline and plated on 96-well black plates at 2 × 10^4^ cells/well. Fluorescence was measured by Infinite F500 (Tecan, Männedorf, Switzerland) using excitation filters 485/20 (DCFDA) or 535/25 (MitoSOX Red) and emission filters 535/25 (DCFDA) or 590/20 (MitoSOX Red).

### 4.7. Mitophagy Detection

Mitophagy staining was performed by incubating the cells at 48 h after irradiation with 100 nM Mitophagy Dye (Mitophagy Detection Kit, Dojindo, Kuma-moto, Japan) for 30 min. Nuclei were concomitantly counterstained with 5 µg/mL Hoechst 33342 (Thermo Fisher Scientific, Waltham, MA, USA). Images were obtained with a fluorescent microscope (Olympus, Tokyo, Japan).

LC3 colocalization in mitochondria was visualized by MitoTracker Deep Red (200 nM for 30 min; Thermo Fisher Scientific, Waltham, MA, USA) staining of cells transfected with pmCherry-LC3 [64] by Viofectin (Viogen, New Taipei City, Taiwan). Images were obtained with a confocal microscope (Olympus, Tokyo, Japan) 48 h after irradiation.

### 4.8. Statistical Analysis

Three or more independent samples for each experiment were analyzed, and all experimental values were expressed as the mean ± standard deviation. The differences between the two groups were assessed by Student’s *t*-test. Multiple comparisons were made by Dunnett’s and Tukey’s tests, as indicated. *p* < 0.05 was considered statistically significant.

## 5. Conclusions

The lncRNA NEAT1v1 confers radioresistance to HCC cells by inducing PINK1/parkin-mediated mitophagy, in which SOD2 and GABARAP are involved (Figure 5B). This study highlights the unprecedented roles of NEAT1v1 in connecting radioresistance and mitophagy in HCC. This finding deepens the understanding of the radioresistance mechanism and provides new insights for developing and improving HCC radiotherapy.

## Data Availability

Data are contained within the article and Supplementary Materials.

## Supplementary Materials

The supporting information can be downloaded at: https://www.mdpi.com/article/10.3390/ijms232214397/s1.

## References

[1] Sung H., Ferlay J., Siegel R.L., Laversanne M., Soerjomataram I., Jemal A., Bray F. (2021). Global cancer statistics 2020: GLOBOCAN estimates of incidence and mortality worldwide for 36 cancers in 185 countries. CA Cancer J. Clin..

[2] Llovet J.M., Pinyol R., Kelley R.K., El-Khoueiry A., Reeves H.L., Wang X.W., Gores G.J., Villanueva A. (2022). Molecular pathogenesis and systemic therapies for hepatocellular carcinoma. Nat. Cancer.

[3] Kamimura K., Terai S. (2021). The promise of radiotherapy for hepatocellular carcinoma. Hepatol. Res..

[4] Su T.S., Liang P., Lu H.Z., Liang J., Gao Y.C., Zhou Y., Huang Y., Tang M.Y., Liang J.N. (2016). Stereotactic body radiation therapy for small primary or recurrent hepatocellular carcinoma in 132 Chinese patients. J. Surg. Oncol..

[5] Shibuya K., Katoh H., Koyama Y., Shiba S., Okamoto M., Okazaki S., Araki K., Kakizaki S., Shirabe K., Ohno T. (2021). Efficacy and Safety of 4 Fractions of Carbon-Ion Radiation Therapy for Hepatocellular Carcinoma: A Prospective Study. Liver Cancer.

[6] Nakagawa S., Naganuma T., Shioi G., Hirose T. (2011). Paraspeckles are subpopulation-specific nuclear bodies that are not essential in mice. J. Cell Biol..

[7] Lin Y., Schmidt B.F., Bruchez M.P., McManus C.J. (2018). Structural analyses of NEAT1 lncRNAs suggest long-range RNA interactions that may contribute to paraspeckle architecture. Nucleic Acids Res..

[8] Koyama S., Tsuchiya H., Amisaki M., Sakaguchi H., Honjo S., Fujiwara Y., Shiota G. (2020). NEAT1 is Required for the Expression of the Liver Cancer Stem Cell Marker CD44. Int. J. Mol. Sci..

[9] Tsuchiya H., Shiota G. (2021). Clinical and Biological Implications of Cancer Stem Cells in Hepatocellular Carcinoma. Yonago Acta Med..

[10] Sakaguchi H., Tsuchiya H., Kitagawa Y., Tanino T., Yoshida K., Uchida N., Shiota G. (2022). NEAT1 Confers Radioresistance to Hepatocellular Carcinoma Cells by Inducing Autophagy through GABARAP. Int. J. Mol. Sci..

[11] Johansen T., Lamark T. (2020). Selective Autophagy: ATG8 Family Proteins, LIR Motifs and Cargo Receptors. J. Mol. Biol..

[12] Nguyen T.N., Padman B.S., Usher J., Oorschot V., Ramm G., Lazarou M. (2016). Atg8 family LC3/GABARAP proteins are crucial for autophagosome-lysosome fusion but not autophagosome formation during PINK1/Parkin mitophagy and starvation. J. Cell Biol..

[13] Ichimura Y., Komatsu M. (2018). Activation of p62/SQSTM1-Keap1-Nuclear Factor Erythroid 2-Related Factor 2 Pathway in Cancer. Front. Oncol..

[14] Takamura A., Komatsu M., Hara T., Sakamoto A., Kishi C., Waguri S., Eishi Y., Hino O., Tanaka K., Mizushima N. (2011). Autophagy-deficient mice develop multiple liver tumors. Genes Dev..

[15] Lin Z., Niu Y., Wan A., Chen D., Liang H., Chen X., Sun L., Zhan S., Chen L., Cheng C. (2020). RNA m6 A methylation regulates sorafenib resistance in liver cancer through FOXO_3_-mediated autophagy. EMBO J..

[16] Shimizu S., Takehara T., Hikita H., Kodama T., Tsunematsu H., Miyagi T., Hosui A., Ishida H., Tatsumi T., Kanto T. (2012). Inhibition of autophagy potentiates the antitumor effect of the multikinase inhibitor sorafenib in hepatocellular carcinoma. Int. J. Cancer.

[17] Amaravadi R.K., Kimmelman A.C., Debnath J. (2019). Targeting Autophagy in Cancer: Recent Advances and Future Directions. Cancer Discov..

[18] Kawamura K., Qi F., Kobayashi J. (2018). Potential relationship between the biological effects of low-dose irradiation and mitochondrial ROS production. J. Radiat. Res..

[19] Vara-Perez M., Felipe-Abrio B., Agostinis P. (2019). Mitophagy in Cancer: A Tale of Adaptation. Cells.

[20] Narendra D.P., Jin S.M., Tanaka A., Suen D.F., Gautier C.A., Shen J., Cookson M.R., Youle R.J. (2010). PINK1 is selectively stabilized on impaired mitochondria to activate Parkin. PLoS Biol..

[21] Narendra D., Tanaka A., Suen D.F., Youle R.J. (2008). Parkin is recruited selectively to impaired mitochondria and promotes their autophagy. J. Cell Biol..

[22] Koyano F., Okatsu K., Kosako H., Tamura Y., Go E., Kimura M., Kimura Y., Tsuchiya H., Yoshihara H., Hirokawa T. (2014). Ubiquitin is phosphorylated by PINK1 to activate parkin. Nature.

[23] Lazarou M., Sliter D.A., Kane L.A., Sarraf S.A., Wang C., Burman J.L., Sideris D.P., Fogel A.I., Youle R.J. (2015). The ubiquitin kinase PINK1 recruits autophagy receptors to induce mitophagy. Nature.

[24] Wong Y.C., Holzbaur E.L. (2014). Optineurin is an autophagy receptor for damaged mitochondria in parkin-mediated mitophagy that is disrupted by an ALS-linked mutation. Proc. Natl. Acad. Sci. USA.

[25] Yu Y., Liu H., Ikeda Y., Amiot B.P., Rinaldo P., Duncan S.A., Nyberg S.L. (2012). Hepatocyte-like cells differentiated from human induced pluripotent stem cells: Relevance to cellular therapies. Stem Cell Res..

[26] Kameyama K., Motoyama K., Tanaka N., Yamashita Y., Higashi T., Arima H. (2017). Induction of mitophagy-mediated antitumor activity with folate-appended methyl-β-cyclodextrin. Int. J. Nanomed..

[27] McWilliams T.G., Prescott A.R., Allen G.F., Tamjar J., Munson M.J., Thomson C., Muqit M.M., Ganley I.G. (2016). mito-QC illuminates mitophagy and mitochondrial architecture in vivo. J. Cell Biol..

[28] Katayama H., Kogure T., Mizushima N., Yoshimori T., Miyawaki A. (2011). A sensitive and quantitative technique for detecting autophagic events based on lysosomal delivery. Chem. Biol..

[29] Xie Y., Liu J., Kang R., Tang D. (2020). Mitophagy Receptors in Tumor Biology. Front. Cell Dev. Biol..

[30] Jin X., Zheng X., Li F., Liu B., Li H., Hirayama R., Li P., Liu X., Shen G., Li Q. (2018). Fragmentation level determines mitochondrial damage response and subsequently the fate of cancer cells exposed to carbon ions. Radiother. Oncol..

[31] Sia J., Szmyd R., Hau E., Gee H.E. (2020). Molecular Mechanisms of Radiation-Induced Cancer Cell Death: A Primer. Front. Cell Dev. Biol..

[32] Liu F., Ai F.Y., Zhang D.C., Tian L., Yang Z.Y., Liu S.J. (2020). LncRNA NEAT1 knockdown attenuates autophagy to elevate 5-FU sensitivity in colorectal cancer via targeting miR-34a. Cancer Med..

[33] Li X., Zhou Y., Yang L., Ma Y., Peng X., Yang S., Li H., Liu J. (2020). LncRNA NEAT1 promotes autophagy via regulating miR-204/ATG3 and enhanced cell resistance to sorafenib in hepatocellular carcinoma. J. Cell Physiol..

[34] Lin Q., Zhang C.F., Guo J.L., Su J.L., Guo Z.K., Li H.Y. (2022). Involvement of NEAT1/PINK1-mediated mitophagy in chronic obstructive pulmonary disease induced by cigarette smoke or PM2.5. Ann. Transl. Med..

[35] Huang Z., Zhao J., Wang W., Zhou J., Zhang J. (2020). Depletion of LncRNA NEAT1 Rescues Mitochondrial Dysfunction Through NEDD4L-Dependent PINK1 Degradation in Animal Models of Alzheimer’s Disease. Front. Cell Neurosci..

[36] Yang D.Y., Zhou X., Liu Z.W., Xu X.Q., Liu C. (2021). LncRNA NEAT1 accelerates renal tubular epithelial cell damage by modulating mitophagy via miR-150-5p-DRP1 axis in diabetic nephropathy. Exp. Physiol..

[37] Salah F.S., Ebbinghaus M., Muley V.Y., Zhou Z., Al-Saadi K.R., Pacyna-Gengelbach M., O’Sullivan G.A., Betz H., König R., Wang Z.Q. (2016). Tumor suppression in mice lacking GABARAP, an Atg8/LC3 family member implicated in autophagy, is associated with alterations in cytokine secretion and cell death. Cell Death Dis..

[38] Miao Y., Zhang Y., Chen Y., Chen L., Wang F. (2010). GABARAP is overexpressed in colorectal carcinoma and correlates with shortened patient survival. Hepatogastroenterology.

[39] Bortnik S., Tessier-Cloutier B., Leung S., Xu J., Asleh K., Burugu S., Magrill J., Greening K., Derakhshan F., Yip S. (2020). Differential expression and prognostic relevance of autophagy-related markers ATG4B, GABARAP, and LC3B in breast cancer. Breast Cancer Res. Treat..

[40] Moussay E., Kaoma T., Baginska J., Muller A., Van Moer K., Nicot N., Nazarov P.V., Vallar L., Chouaib S., Berchem G. (2011). The acquisition of resistance to TNFα in breast cancer cells is associated with constitutive activation of autophagy as revealed by a transcriptome analysis using a custom microarray. Autophagy.

[41] Liu Y., Wang D., Lei M., Gao J., Cui Y., Jin X., Yu Q., Jiang Y., Guo Y., Liu Y. (2021). GABARAP suppresses EMT and breast cancer progression via the AKT/mTOR signaling pathway. Aging.

[42] Kim E.H., Sohn S., Kwon H.J., Kim S.U., Kim M.J., Lee S.J., Choi K.S. (2007). Sodium selenite induces superoxide-mediated mitochondrial damage and subsequent autophagic cell death in malignant glioma cells. Cancer Res..

[43] Zhou R., Yazdi A.S., Menu P., Tschopp J. (2011). A role for mitochondria in NLRP3 inflammasome activation. Nature.

[44] Lee J., Giordano S., Zhang J. (2012). Autophagy, mitochondria and oxidative stress: Cross-talk and redox signalling. Biochem. J..

[45] Yao N., Wang C., Hu N., Li Y., Liu M., Lei Y., Chen M., Chen L., Chen C., Lan P. (2019). Inhibition of PINK1/Parkin-dependent mitophagy sensitizes multidrug-resistant cancer cells to B5G1, a new betulinic acid analog. Cell Death Dis..

[46] Rubio N., Coupienne I., Di Valentin E., Heirman I., Grooten J., Piette J., Agostinis P. (2012). Spatiotemporal autophagic degradation of oxidatively damaged organelles after photodynamic stress is amplified by mitochondrial reactive oxygen species. Autophagy.

[47] Chen G.D., Zhang J.L., Chen Y.T., Zhang J.X., Wang T., Zeng Q.Y. (2018). Insulin alleviates mitochondrial oxidative stress involving upregulation of superoxide dismutase 2 and uncoupling protein 2 in septic acute kidney injury. Exp. Ther. Med..

[48] Dhar S.K., Batinic-Haberle I., St Clair D.K. (2019). UVB-induced inactivation of manganese-containing superoxide dismutase promotes mitophagy via ROS-mediated mTORC2 pathway activation. J. Biol. Chem..

[49] Li Y., Ma Y., Song L., Yu L., Zhang L., Zhang Y., Xing Y., Yin Y., Ma H. (2018). SIRT3 deficiency exacerbates p53/Parkin-mediated mitophagy inhibition and promotes mitochondrial dysfunction: Implication for aged hearts. Int. J. Mol. Med..

[50] Dongil P., Pérez-García A., Hurtado-Carneiro V., Herrero-de-Dios C., Blazquez E., Alvarez E., Sanz C. (2018). Pas Kinase Deficiency Triggers Antioxidant Mechanisms in the Liver. Sci. Rep..

[51] Xi J., Rong Y., Zhao Z., Huang Y., Wang P., Luan H., Xing Y., Li S., Liao J., Dai Y. (2021). Scutellarin ameliorates high glucose-induced vascular endothelial cells injury by activating PINK1/Parkin-mediated mitophagy. J. Ethnopharmacol..

[52] Ryoo I.G., Kwak M.K. (2018). Regulatory crosstalk between the oxidative stress-related transcription factor Nfe2l2/Nrf2 and mitochondria. Toxicol. Appl. Pharmacol..

[53] Sheik Abdul N., Nagiah S., Chuturgoon A.A. (2019). Fusaric acid induces NRF2 as a cytoprotective response to prevent NLRP3 activation in the liver derived HepG2 cell line. Toxicol. In Vitro.

[54] Bin J., Bai T., Zhao Q., Duan X., Deng S., Xu Y. (2021). Parkin overexpression reduces inflammation-mediated cardiomyocyte apoptosis through activating Nrf2/ARE signaling pathway. J. Recept. Signal Transduct. Res..

[55] Alves A.F., Moura A.C., Andreolla H.F., Veiga A.B.G.D., Fiegenbaum M., Giovenardi M., Almeida S. (2021). Gene expression evaluation of antioxidant enzymes in patients with hepatocellular carcinoma: RT-qPCR and bioinformatic analyses. Genet. Mol. Biol..

[56] Hempel N., Carrico P.M., Melendez J.A. (2011). Manganese superoxide dismutase (Sod2) and redox-control of signaling events that drive metastasis. Anticancer Agents Med. Chem..

[57] Yuan Y., Cao W., Zhou H., Qian H., Wang H. (2021). CLTRN, Regulated by NRF1/RAN/DLD Protein Complex, Enhances Radiation Sensitivity of Hepatocellular Carcinoma Cells Through Ferroptosis Pathway. Int. J. Radiat. Oncol. Biol. Phys..

[58] Lan X., Wu Y.Z., Wang Y., Wu F.R., Zang C.B., Tang C., Cao S., Li S.L. (2013). CD133 silencing inhibits stemness properties and enhances chemoradiosensitivity in CD133-positive liver cancer stem cells. Int. J. Mol. Med..

[59] Chen C.C., Chen C.Y., Wang S.H., Yeh C.T., Su S.C., Ueng S.H., Chuang W.Y., Hsueh C., Wang T.H. (2018). Melatonin Sensitizes Hepatocellular Carcinoma Cells to Chemotherapy Through Long Non-Coding RNA RAD51-AS1-Mediated Suppression of DNA Repair. Cancers.

[60] Wang J., Zhao H., Yu J., Xu X., Jing H., Li N., Tang Y., Wang S., Li Y., Cai J. (2021). MiR-320b/RAD21 axis affects hepatocellular carcinoma radiosensitivity to ionizing radiation treatment through DNA damage repair signaling. Cancer Sci..

[61] Hu L., Wang H., Huang L., Zhao Y., Wang J. (2016). The Protective Roles of ROS-Mediated Mitophagy on 125I Seeds Radiation Induced Cell Death in HCT116 Cells. Oxidative Med. Cell Longev..

[62] Chang M., Song X., Geng X., Wang X., Wang W., Chen T.C., Xie L., Song X. (2018). Temozolomide-Perillyl alcohol conjugate impairs Mitophagy flux by inducing lysosomal dysfunction in non-small cell lung Cancer cells and sensitizes them to irradiation. J. Exp. Clin. Cancer Res..

[63] Gallaher S.D., Berk A.J. (2013). A rapid Q-PCR titration protocol for adenovirus and helper-dependent adenovirus vectors that produces biologically relevant results. J. Virol. Methods.

[64] Miki T., Awa M., Nishikawa Y., Kiyonaka S., Wakabayashi M., Ishihama Y., Hamachi I. (2016). A conditional proteomics approach to identify proteins involved in zinc homeostasis. Nat. Methods.

