# Supplementary material for: NEAT1 Confers Radioresistance to Hepatocellular Carcinoma Cells by Inducing PINK1/Parkin-Mediated Mitophagy
Source: Int J Mol Sci. 2022 Nov 19;23(22):14397. doi: 10.3390/ijms232214397 (PMC9692527; doi:10.3390/ijms232214397)
Supplement: Supplementary file 1 [file ijms-23-14397-s001.zip › FigureS1-S3.pdf]

Figure S1

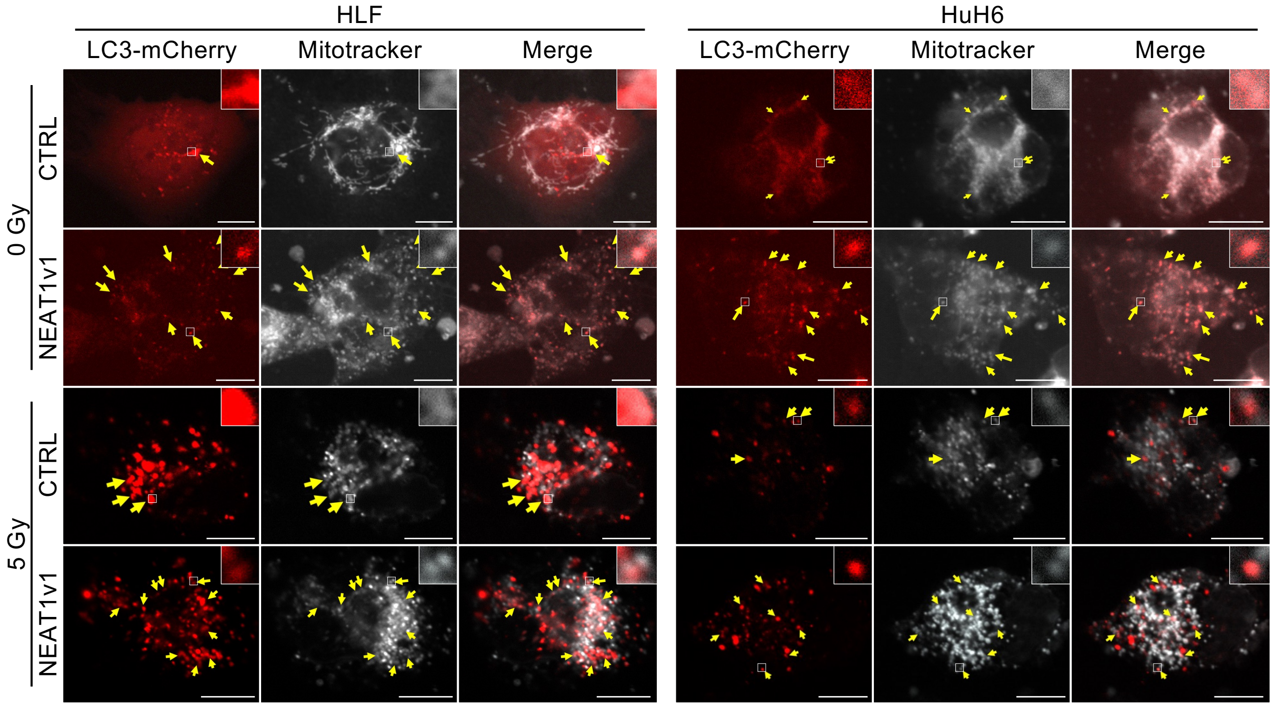

Figure S1. Representative images of colocalization of mitochondria with LC3. Control (CTRL) and NEAT1v1-overexpressing HCC cell lines (HLF and HuH6) were transfected with a LC3-mCherry-expressing vector. Following irradiation at 0 or 5 Gy, the cells were stained with Mitotracker Deep Red. The yellow arrow indicates mitochondria colocalized with LC3-mCherry. The white box marks the area enlarged in the inset.

Figure S2

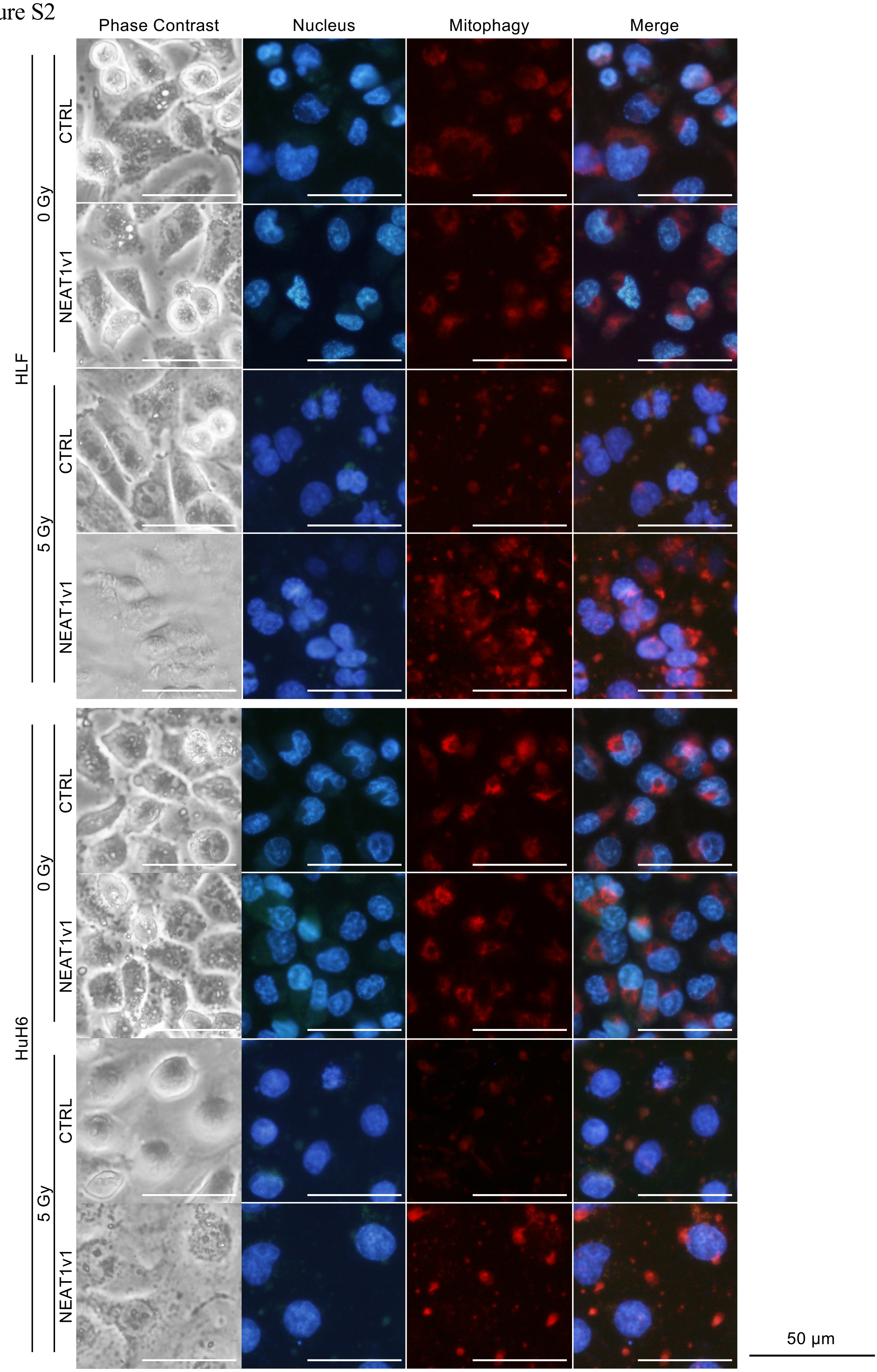

Figure S2. High magnification images of Figure 2A.

Figure S3

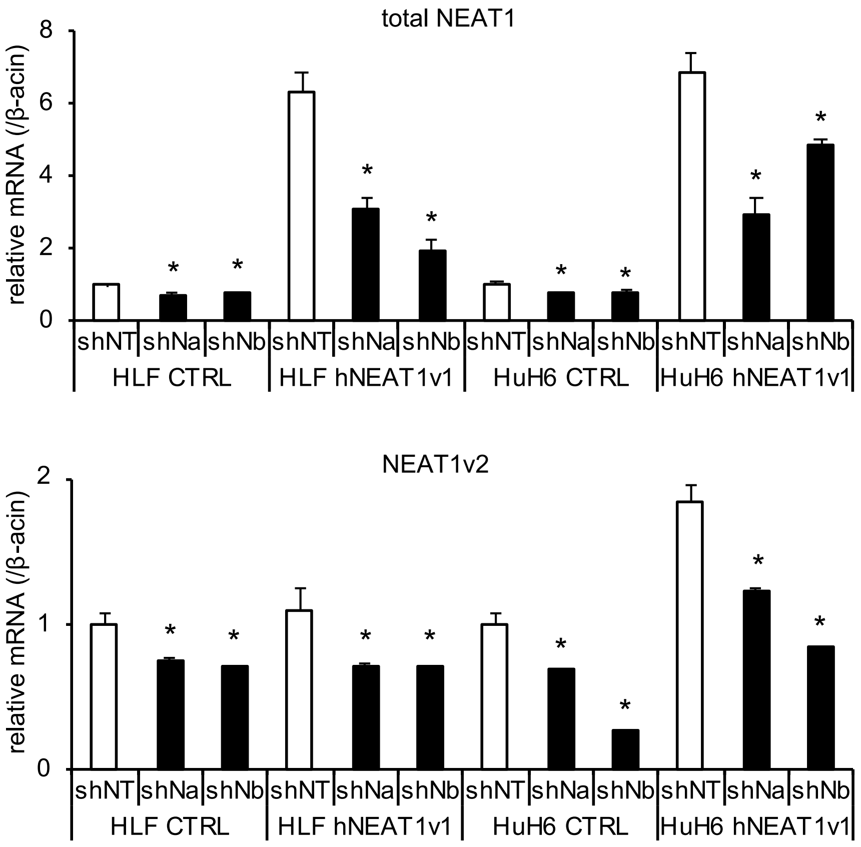

Figure S3. Confirmation of NEAT1 knockdown effciency. Expression levels of NEAT1 in control (CTRL) or NEAT1v1-overexpressing cells transduced with adenoviruses expressing non-target shRNA (shNT), or NEAT1-specific shRNAs (shNa and shNb). *, $P < 0.05$ (Dunnett's test, vs. shNT; n = 3)
